# Potassium morpholine-4-carbodithio­ate monohydrate

**DOI:** 10.1107/S1600536812029613

**Published:** 2012-07-04

**Authors:** Ana C. Mafud

**Affiliations:** a Instituto de Física de São Carlos, Universidade de São Paulo, Av. Trabalhador Sãocarlense, 400, Caixa Postal 369, 13566-590 São Carlos, SP, Brazil

## Abstract

In the ionic title compound, K^+^·C_5_H_8_NOS_2_^−^·H_2_O, the morpholine ring of the morpholine-4-carbodithio­ate anion has a chair conformation. The potassium cation is coordinated by four S and four O atoms in a bipyramidal reversed geometry. In the crystal, the three components are linked, generating infinite two-dimensional networks that lie parallel to the *bc* plane. These layers are linked *via* O—H⋯S hydrogen bonds, forming a three-dimensional structure.

## Related literature

For the crystal structures of similar compounds, see: Oskarsson *et al.* (1979 ▶); Albertsson *et al.* (1980 ▶); Ymén (1982 ▶); Mafud & Gambardella (2011*a* ▶,*b* ▶); Mafud *et al.* (2011 ▶). For puckering parameters, see: Cremer & Pople (1975 ▶).
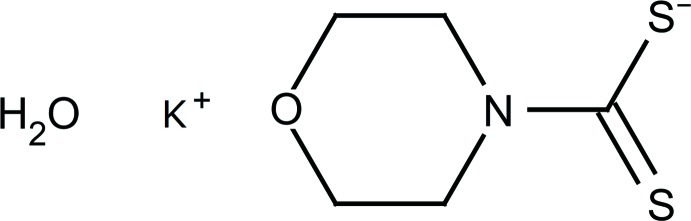


## Experimental

### 

#### Crystal data

K^+^·C_5_H_8_NOS_2_^−^·H_2_O
*M* *_r_* = 219.36
Monoclinic, 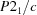

*a* = 6.7235 (10) Å
*b* = 17.260 (4) Å
*c* = 8.1904 (10) Å
β = 108.994 (10)°
*V* = 898.7 (3) Å^3^
*Z* = 4
Mo *K*α radiation
μ = 1.01 mm^−1^
*T* = 290 K
0.45 × 0.30 × 0.20 mm

#### Data collection

Enraf–Nonius TurboCAD-4 diffractometer
Absorption correction: refined from Δ*F* (Walker & Stuart, 1983 ▶) *T*_min_ = 0.512, *T*_max_ = 0.818
2779 measured reflections
2618 independent reflections
1615 reflections with *I* > 2σ(*I*)
*R*_int_ = 0.027
3 standard reflections every 120 min intensity decay: 10%

#### Refinement

*R*[*F* ^2^ > 2σ(*F* ^2^)] = 0.047
*wR*(*F* ^2^) = 0.130
*S* = 1.01
2618 reflections
106 parameters
3 restraints
H atoms treated by a mixture of independent and constrained refinement
Δρ_max_ = 0.50 e Å^−3^
Δρ_min_ = −0.61 e Å^−3^

### 

Data collection: *CAD-4 EXPRESS* (Enraf–Nonius, 1994 ▶); cell refinement: *CAD-4 EXPRESS*; data reduction: *XCAD4* (Harms & Wocadlo, 1995 ▶); program(s) used to solve structure: *SIR92* (Altomare *et al.*, 1994 ▶); program(s) used to refine structure: *SHELXL97* (Sheldrick, 2008 ▶); molecular graphics: *ORTEP-3 for Windows* (Farrugia, 1997 ▶); software used to prepare material for publication: *WinGX* (Farrugia, 1999 ▶).

## Supplementary Material

Crystal structure: contains datablock(s) I, global. DOI: 10.1107/S1600536812029613/su2446sup1.cif

Structure factors: contains datablock(s) I. DOI: 10.1107/S1600536812029613/su2446Isup2.hkl

Supplementary material file. DOI: 10.1107/S1600536812029613/su2446Isup3.cml

Additional supplementary materials:  crystallographic information; 3D view; checkCIF report

## Figures and Tables

**Table 1:** Hydrogen-bond geometry (Å, °)

*D*—H⋯*A*	*D*—H	H⋯*A*	*D*⋯*A*	*D*—H⋯*A*
O2—H1*O*⋯S1^i^	0.86 (4)	2.45 (4)	3.219 (3)	149 (3)
O2—H2*O*⋯S1^ii^	0.85 (3)	2.87 (5)	3.462 (3)	129 (4)

Symmetry codes: (i) 
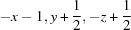
; (ii) 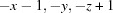.

**Fig. 1.**
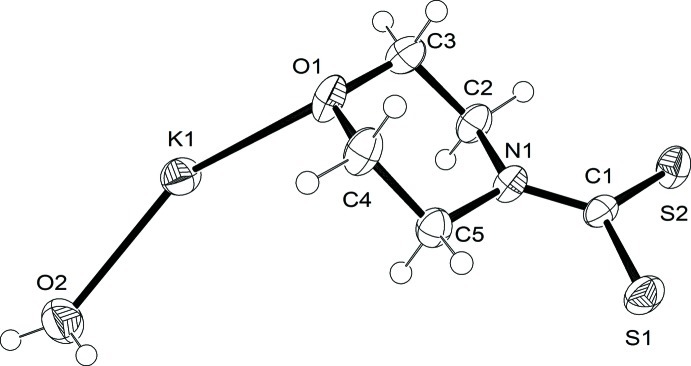
A view of the molecular structure of the asymmetric unit of the title compound, with the numbering scheme. The displacement ellipsoids are drawn at the 50% probability level.

**Fig. 2.**
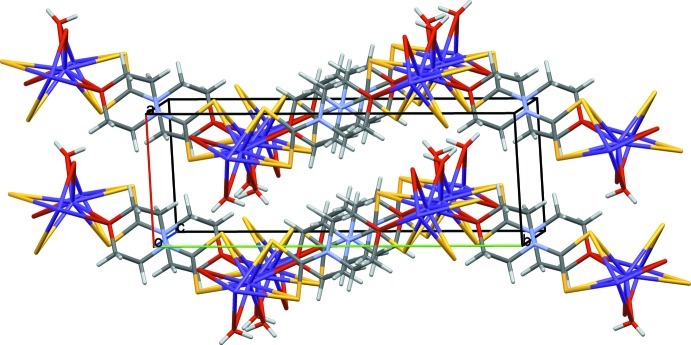
The view along the c axis of the crystal packing of the title compound.

## supplementary crystallographic information

### Comment

The title compound, Fig. 1, is composed of a morpholinedithiocarbamate anion in contact with a potassium cation, which in turn is linked with a water molecule of crystallization. The crystal structure of similar compounds, for example Sodium 1-*R*-carbodithioate dihydrate, have been reported (Oskarsson *et al.*, 1979; Albertsson *et al.*, 1980; Ymén, 1982; Mafud & Gambardella *et al.*, 2011*a*,*b*).

The six-membered morpholine ring has a chair conformation with Puckering parameters [Cremer & Pople, 1975)] Q = 0.548 (3) Å, θ = 173,3(3)°, φ2 = 2,6(3,4)°.

In the crystal, a polymeric structure is built by coordination of the potassium cation to four sulfur [K···S = 3.2670 (13) - 3.3797 (14) Å] and four oxygen [K···O = 2.828 (3) - 3.007 (3) Å] atoms, with a bi-pyramidal reversed geometry. This configuration generates close packed layers which remain cohesive in crystal stacking by van der Waals interactions. The distances of these contacts are slightly less than the sum of the van der Waals radii.

The crystal packing gives rise to a supramolecular structure, whose infinite two-dimensional network lies parallel to the bc plane (Fig. 2). These layers are linked via O-H···S hydrogen bonds (Table 1) to form a three-dimensional structure.

### Experimental

The potassium salts of DTC were prepared by direct reaction between amine and carbon disulfide (CS_2_) in the presence of a stoichiometric amount of potassium hydroxide in ethanol/water 1:1 (*v*:*v*). The reaction mixture was placed in the freezer for 12 h and then filtered through a Büchner funnel, washed with cold ether and the product recrystallized in an ethanol-water mixture 1:1 (*v*:*v*). The obtained solid was recrystallized from ethanol-water 1:1 (*v*/*v*) and dried in a vacuum oven at 323 K for 8 h. Colourless crystals, suitable for X-ray diffraction analysis, were obtained. On heating they sublimed and decomposed.

### Refinement

The H-atom positions of the water molecule were located in a difference Fourier map and were refined with U_iso_(H) = 1.5U_eq_(O); O—H = 0.86 (4) and 0.85 (3) Å. The C-bound H-atoms of the anion were included in calculated positions and treated as riding atoms: C—H = 0.97 Å, with *U*_iso_(H) = 1.2*U*_eq_(parent C-atom).

### Crystal data

**Table:**

K^+^·C_5_H_8_NOS_2_^−^·H_2_O	*F*(000) = 456
*M**_r_* = 219.36	*D*_x_ = 1.621 Mg m^−^^3^
Monoclinic, *P*2_1_/*c*	Mo *K*α radiation, λ = 0.71073 Å
Hall symbol: -P 2ybc	Cell parameters from 16 reflections
*a* = 6.7235 (10) Å	θ = 9.8–18.3°
*b* = 17.260 (4) Å	µ = 1.01 mm^−^^1^
*c* = 8.1904 (10) Å	*T* = 290 K
β = 108.994 (10)°	Prism, colourless
*V* = 898.7 (3) Å^3^	0.45 × 0.3 × 0.2 mm
*Z* = 4	

### Data collection

**Table:**

Enraf–Nonius TurboCAD-4 diffractometer	1615 reflections with *I* > 2σ(*I*)
Radiation source: Enraf Nonius FR590	*R*_int_ = 0.027
Graphite monochromator	θ_max_ = 30.0°, θ_min_ = 2.4°
non–profiled ω/2θ scans	*h* = −9→8
Absorption correction: part of the refinement model (Δ*F*) (Walker & Stuart, 1983)	*k* = 0→24
*T*_min_ = 0.512, *T*_max_ = 0.818	*l* = 0→11
2779 measured reflections	3 standard reflections every 120 min
2618 independent reflections	intensity decay: 10%

### Refinement

**Table:**

Refinement on *F*^2^	Primary atom site location: structure-invariant direct methods
Least-squares matrix: full	Secondary atom site location: difference Fourier map
*R*[*F*^2^ > 2σ(*F*^2^)] = 0.047	Hydrogen site location: inferred from neighbouring sites
*wR*(*F*^2^) = 0.130	H atoms treated by a mixture of independent and constrained refinement
*S* = 1.01	*w* = 1/[σ^2^(*F*_o_^2^) + (0.0644*P*)^2^] where *P* = (*F*_o_^2^ + 2*F*_c_^2^)/3
2618 reflections	(Δ/σ)_max_ < 0.001
106 parameters	Δρ_max_ = 0.50 e Å^−^^3^
3 restraints	Δρ_min_ = −0.61 e Å^−^^3^

### Special details

**Table:**

Geometry. Bond distances, angles etc. have been calculated using the rounded fractional coordinates. All su's are estimated from the variances of the (full) variance-covariance matrix. The cell esds are taken into account in the estimation of distances, angles and torsion angles
Refinement. Refinement of *F*^2^ against ALL reflections. The weighted *R*-factor *wR* and goodness of fit *S* are based on *F*^2^, conventional *R*-factors *R* are based on *F*, with *F* set to zero for negative *F*^2^. The threshold expression of *F*^2^ > σ(*F*^2^) is used only for calculating *R*-factors(gt) *etc*. and is not relevant to the choice of reflections for refinement. *R*-factors based on *F*^2^ are statistically about twice as large as those based on *F*, and *R*- factors based on ALL data will be even larger.

### Fractional atomic coordinates and isotropic or equivalent isotropic displacement parameters (Å^2^)

**Table:**

	*x*	*y*	*z*	*U*_iso_*/*U*_eq_	
K1	−0.28085 (12)	0.24637 (4)	0.48217 (9)	0.0366 (2)	
S1	−0.06278 (13)	−0.16683 (4)	0.69255 (11)	0.0320 (2)	
S2	0.36659 (13)	−0.09853 (4)	0.80129 (12)	0.0336 (3)	
O1	−0.1501 (4)	0.13219 (12)	0.7743 (3)	0.0366 (7)	
O2	−0.5955 (4)	0.27515 (16)	0.1617 (4)	0.0496 (9)	
N1	0.0172 (4)	−0.01514 (13)	0.7295 (3)	0.0261 (7)	
C1	0.1003 (5)	−0.08662 (15)	0.7399 (4)	0.0240 (8)	
C2	0.1460 (5)	0.05521 (16)	0.7583 (4)	0.0293 (9)	
C3	0.0660 (5)	0.11438 (18)	0.8574 (4)	0.0334 (10)	
C4	−0.2719 (5)	0.06303 (18)	0.7624 (5)	0.0348 (10)	
C5	−0.2096 (4)	0.00131 (17)	0.6581 (4)	0.0289 (8)	
H1O	−0.682 (6)	0.3070 (18)	0.093 (5)	0.0740*	
H2A	0.14220	0.07690	0.64810	0.0350*	
H2B	0.29080	0.04230	0.82250	0.0350*	
H2O	−0.645 (7)	0.2294 (12)	0.154 (6)	0.0740*	
H3A	0.08450	0.09460	0.97230	0.0400*	
H3B	0.14850	0.16140	0.86940	0.0400*	
H4A	−0.41980	0.07520	0.70910	0.0420*	
H4B	−0.25230	0.04350	0.87770	0.0420*	
H5A	−0.28850	−0.04570	0.65890	0.0350*	
H5B	−0.24380	0.01850	0.53940	0.0350*	

### Atomic displacement parameters (Å^2^)

**Table:**

	*U*^11^	*U*^22^	*U*^33^	*U*^12^	*U*^13^	*U*^23^
K1	0.0408 (4)	0.0356 (4)	0.0341 (4)	−0.0006 (3)	0.0130 (3)	−0.0026 (3)
S1	0.0383 (4)	0.0207 (3)	0.0366 (4)	−0.0060 (3)	0.0116 (3)	−0.0032 (3)
S2	0.0297 (4)	0.0270 (4)	0.0454 (5)	0.0033 (3)	0.0141 (3)	−0.0004 (3)
O1	0.0370 (12)	0.0220 (10)	0.0551 (15)	0.0040 (9)	0.0210 (11)	−0.0031 (10)
O2	0.0444 (15)	0.0393 (14)	0.0546 (17)	0.0006 (12)	0.0018 (13)	0.0048 (13)
N1	0.0270 (12)	0.0204 (11)	0.0339 (14)	−0.0009 (9)	0.0142 (10)	−0.0035 (10)
C1	0.0313 (14)	0.0203 (13)	0.0231 (14)	−0.0011 (11)	0.0124 (11)	−0.0031 (11)
C2	0.0310 (15)	0.0205 (13)	0.0400 (18)	−0.0044 (11)	0.0164 (14)	−0.0021 (12)
C3	0.0356 (17)	0.0278 (15)	0.0381 (18)	−0.0040 (12)	0.0140 (14)	−0.0072 (13)
C4	0.0326 (16)	0.0267 (15)	0.051 (2)	0.0004 (12)	0.0218 (15)	−0.0025 (14)
C5	0.0267 (14)	0.0255 (14)	0.0349 (16)	−0.0005 (11)	0.0108 (13)	−0.0004 (12)

### Geometric parameters (Å, º)

**Table:**

K1—O1	3.002 (2)	N1—C2	1.465 (4)
K1—O2	2.828 (3)	N1—C1	1.346 (4)
K1—S1^i^	3.2670 (13)	N1—C5	1.472 (4)
K1—S2^i^	3.3630 (13)	C2—C3	1.508 (4)
K1—S1^ii^	3.3797 (14)	C4—C5	1.508 (5)
K1—S2^ii^	3.3708 (13)	C2—H2A	0.9700
K1—O1^iii^	3.007 (3)	C2—H2B	0.9700
K1—O2^iv^	2.967 (3)	C3—H3A	0.9700
S1—C1	1.730 (3)	C3—H3B	0.9700
S2—C1	1.707 (4)	C4—H4A	0.9700
O1—C3	1.423 (4)	C4—H4B	0.9700
O1—C4	1.433 (4)	C5—H5A	0.9700
O2—H2O	0.85 (3)	C5—H5B	0.9700
O2—H1O	0.86 (4)		
			
O1—K1—O2	142.34 (8)	K1^iv^—O1—C3	108.72 (17)
S1^i^—K1—O1	72.85 (5)	K1^iv^—O1—C4	110.7 (2)
S2^i^—K1—O1	99.11 (5)	K1—O2—K1^iii^	89.96 (8)
S1^ii^—K1—O1	90.44 (6)	K1^iii^—O2—H2O	100 (3)
S2^ii^—K1—O1	89.55 (5)	K1—O2—H2O	94 (3)
O1—K1—O1^iii^	147.70 (8)	H1O—O2—H2O	112 (4)
O1—K1—O2^iv^	66.04 (8)	K1—O2—H1O	149 (2)
S1^i^—K1—O2	142.49 (6)	K1^iii^—O2—H1O	101 (3)
S2^i^—K1—O2	98.33 (6)	C1—N1—C5	123.9 (3)
S1^ii^—K1—O2	94.95 (6)	C2—N1—C5	112.7 (2)
S2^ii^—K1—O2	65.49 (6)	C1—N1—C2	122.6 (3)
O1^iii^—K1—O2	67.68 (8)	S1—C1—N1	120.0 (3)
O2—K1—O2^iv^	92.48 (9)	S2—C1—N1	120.2 (2)
S1^i^—K1—S2^i^	53.28 (3)	S1—C1—S2	119.81 (16)
S1^i^—K1—S1^ii^	97.59 (3)	N1—C2—C3	110.6 (3)
S1^i^—K1—S2^ii^	145.67 (4)	O1—C3—C2	112.1 (3)
S1^i^—K1—O1^iii^	83.26 (5)	O1—C4—C5	111.7 (3)
S1^i^—K1—O2^iv^	94.72 (6)	N1—C5—C4	110.7 (3)
S1^ii^—K1—S2^i^	143.59 (3)	N1—C2—H2A	110.00
S2^i^—K1—S2^ii^	161.01 (4)	N1—C2—H2B	109.00
S2^i^—K1—O1^iii^	82.85 (5)	C3—C2—H2A	109.00
S2^i^—K1—O2^iv^	64.28 (6)	C3—C2—H2B	110.00
S1^ii^—K1—S2^ii^	52.27 (3)	H2A—C2—H2B	108.00
S1^ii^—K1—O1^iii^	71.17 (5)	O1—C3—H3A	109.00
S1^ii^—K1—O2^iv^	148.77 (6)	O1—C3—H3B	109.00
S2^ii^—K1—O1^iii^	98.84 (5)	C2—C3—H3A	109.00
S2^ii^—K1—O2^iv^	104.89 (6)	C2—C3—H3B	109.00
O1^iii^—K1—O2^iv^	139.03 (8)	H3A—C3—H3B	108.00
K1^v^—S1—C1	87.47 (11)	O1—C4—H4A	109.00
K1^ii^—S1—C1	87.09 (11)	O1—C4—H4B	109.00
K1^v^—S1—K1^ii^	76.09 (3)	C5—C4—H4A	109.00
K1^v^—S2—C1	84.71 (10)	C5—C4—H4B	109.00
K1^ii^—S2—C1	87.73 (10)	H4A—C4—H4B	108.00
K1^v^—S2—K1^ii^	74.95 (2)	N1—C5—H5A	110.00
K1—O1—C3	120.77 (19)	N1—C5—H5B	110.00
K1—O1—C4	118.7 (2)	C4—C5—H5A	109.00
K1—O1—K1^iv^	85.98 (6)	C4—C5—H5B	109.00
C3—O1—C4	108.9 (2)	H5A—C5—H5B	108.00
			
O2—K1—O1—C3	142.4 (2)	O2—K1—S1^ii^—C1^ii^	−72.98 (12)
O2—K1—O1—C4	3.2 (3)	O2—K1—S1^ii^—K1^iii^	15.13 (6)
O2—K1—O1—K1^iv^	−108.20 (12)	O1—K1—S2^ii^—C1^ii^	−71.26 (12)
S1^i^—K1—O1—C3	−54.13 (19)	O1—K1—S2^ii^—K1^iii^	−156.39 (6)
S1^i^—K1—O1—C4	166.7 (2)	O2—K1—S2^ii^—C1^ii^	137.86 (12)
S1^i^—K1—O1—K1^iv^	55.30 (5)	O2—K1—S2^ii^—K1^iii^	52.73 (7)
S2^i^—K1—O1—C3	−101.0 (2)	O1—K1—O1^iii^—K1^iii^	111.73 (12)
S2^i^—K1—O1—C4	119.8 (2)	O1—K1—O1^iii^—C3^iii^	−9.5 (2)
S2^i^—K1—O1—K1^iv^	8.40 (6)	O1—K1—O1^iii^—C4^iii^	−129.1 (2)
S1^ii^—K1—O1—C3	43.7 (2)	O2—K1—O1^iii^—K1^iii^	−50.45 (8)
S1^ii^—K1—O1—C4	−95.5 (2)	O2—K1—O1^iii^—C3^iii^	−171.6 (2)
S1^ii^—K1—O1—K1^iv^	153.11 (5)	O2—K1—O1^iii^—C4^iii^	68.8 (2)
S2^ii^—K1—O1—C3	96.0 (2)	O1—K1—O2^iv^—K1^iv^	52.11 (7)
S2^ii^—K1—O1—C4	−43.2 (2)	O2—K1—O2^iv^—K1^iv^	−159.92 (8)
S2^ii^—K1—O1—K1^iv^	−154.62 (6)	K1^v^—S1—C1—S2	39.50 (18)
O1^iii^—K1—O1—C3	−10.0 (3)	K1^v^—S1—C1—N1	−140.0 (2)
O1^iii^—K1—O1—C4	−149.2 (2)	K1^ii^—S1—C1—S2	−36.69 (18)
O1^iii^—K1—O1—K1^iv^	99.41 (13)	K1^ii^—S1—C1—N1	143.8 (2)
O2^iv^—K1—O1—C3	−157.5 (2)	K1^v^—S2—C1—S1	−38.31 (18)
O2^iv^—K1—O1—C4	63.4 (2)	K1^v^—S2—C1—N1	141.2 (3)
O2^iv^—K1—O1—K1^iv^	−48.08 (8)	K1^ii^—S2—C1—S1	36.78 (18)
O1—K1—O2—K1^iii^	−113.36 (12)	K1^ii^—S2—C1—N1	−143.7 (2)
S1^i^—K1—O2—K1^iii^	93.11 (11)	K1—O1—C3—C2	−81.8 (3)
S2^i^—K1—O2—K1^iii^	129.81 (6)	C4—O1—C3—C2	60.9 (3)
S1^ii^—K1—O2—K1^iii^	−16.20 (6)	K1^iv^—O1—C3—C2	−178.43 (19)
S2^ii^—K1—O2—K1^iii^	−60.59 (5)	K1—O1—C4—C5	82.8 (3)
O1^iii^—K1—O2—K1^iii^	51.11 (7)	C3—O1—C4—C5	−60.7 (3)
O2^iv^—K1—O2—K1^iii^	−165.83 (8)	K1^iv^—O1—C4—C5	179.8 (2)
O1—K1—S1^i^—C1^i^	−136.36 (13)	C2—N1—C1—S1	−176.4 (2)
O1—K1—S1^i^—K1^iv^	−48.73 (6)	C2—N1—C1—S2	4.1 (4)
O2—K1—S1^i^—C1^i^	27.08 (16)	C5—N1—C1—S1	−8.1 (4)
O2—K1—S1^i^—K1^iv^	114.71 (11)	C5—N1—C1—S2	172.4 (2)
O1—K1—S2^i^—C1^i^	81.34 (12)	C1—N1—C2—C3	−140.7 (3)
O1—K1—S2^i^—K1^iv^	−7.73 (6)	C5—N1—C2—C3	49.8 (3)
O2—K1—S2^i^—C1^i^	−132.18 (12)	C1—N1—C5—C4	140.7 (3)
O2—K1—S2^i^—K1^iv^	138.75 (7)	C2—N1—C5—C4	−50.0 (3)
O1—K1—S1^ii^—C1^ii^	69.70 (12)	N1—C2—C3—O1	−55.7 (3)
O1—K1—S1^ii^—K1^iii^	157.81 (5)	O1—C4—C5—N1	55.5 (3)

Symmetry codes: (i) −*x*, *y*+1/2, −*z*+3/2; (ii) −*x*, −*y*, −*z*+1; (iii) *x*, −*y*+1/2, *z*−1/2; (iv) *x*, −*y*+1/2, *z*+1/2; (v) −*x*, *y*−1/2, −*z*+3/2.

### Hydrogen-bond geometry (Å, º)

**Table:**

*D*—H···*A*	*D*—H	H···*A*	*D*···*A*	*D*—H···*A*
O2—H1*O*···S1^vi^	0.86 (4)	2.45 (4)	3.219 (3)	149 (3)
O2—H2*O*···S1^vii^	0.85 (3)	2.87 (5)	3.462 (3)	129 (4)

Symmetry codes: (vi) −*x*−1, *y*+1/2, −*z*+1/2; (vii) −*x*−1, −*y*, −*z*+1.
