# Potassium morpholine-4-carbodithio­ate monohydrate. Corrigendum

**DOI:** 10.1107/S1600536813008994

**Published:** 2013-04-05

**Authors:** Ana C. Mafud, Maria Teresa Prado Gambardella

**Affiliations:** a Instituto de Física de São Carlos, Universidade de São Paulo, Av. Trabalhador Sãocarlense, 400, Caixa Postal 369, 13566-590 São Carlos, SP, Brazil; b Instituto de Química de São Carlos, Universidade de São Paulo, Av. Trabalhador Sãocarlense, 400, Caixa Postal 369, 13566-590 São Carlos, SP, Brazil

## Abstract

The author list in the paper by Mafud [*Acta Cryst.* (2012), E**68**, m1025] is corrected.

## Abstract 2

Corrigendum to *Acta Cryst.* (2012), E**68**, m1025.

In the paper by Mafud (2012) ▶, one of the original authors was omitted. The correct list is given above.

## References

[bb1] Mafud, A. C. (2012). *Acta Cryst.* E**68**, m1025. 10.1107/S1600536812029613 PMC3414100 22904707

